# Soroprevalência de hepatite A em adolescentes escolares: estudo transversal em Curitiba, Paraná, 2024

**DOI:** 10.1590/S2237-96222026v35e20250870.pt

**Published:** 2026-02-09

**Authors:** Janilza Silveira Silva, Renata Barbosa Vilaça Marques de Carvalho, Tatiane Bartneck Telles, Alcides Souto de Oliveira, Leia Regina Silva, Liza Regina Bueno Rosso, Diego Spinoza dos Santos, Clea Elisa Ribeiro, Monique Boese, Katiuska Ferraz Jansen Negrello, Cláudia Weingaertner Palm, Dirlene Pacheco Venâncio, Aroldo José Borges Carneiro, Silvio Luis Rodrigues Almeida

**Affiliations:** 1 Ministério da Saúde, Programa de Treinamento em Epidemiologia Aplicada aos Serviços do SUS, Brasília, DF, Brasil; 2 Prefeitura de Curitiba, Secretaria Municipal da Saúde, Paraná, PR, Brasil; 3 Centro de Epidemiologia da Secretaria Municipal da Saúde de Curitiba, Paraná, PR, Brasil; 4 Centro de Informações Estratégicas em Vigilância em Saúde de Curitiba, Paraná, PR, Brasil

**Keywords:** Hepatites, Vírus da Hepatite A, Adolescente, Surto de Doenças, Estudos Soroepidemiológicos., Hepatitis, Virus de la Hepatitis A, Adolescente, Brotes de Enfermedades, Estudios Seroepidemiológicos.

## Abstract

**Objetivo:**

Estimar a soroprevalência de anticorpos contra o Vírus da Hepatite A (anti-HAV) - Imunoglobulina G (IgG) e Imunoglobulina M (IgM) - em adolescentes escolares de Curitiba, Paraná, no contexto de um surto, e identificar a proporção de infecções naturais e imunidade vacinal.

**Métodos:**

Estudo transversal com base em inquérito sorológico realizado entre 3 e 5 de julho de 2024 em cinco escolas estaduais. A amostragem foi aleatória estratificada proporcional ao total de estudantes por escola. Foram incluídos adolescentes de 10-19 anos com consentimento individual e dos responsáveis legais. A coleta foi realizada nas escolas e as amostras analisadas por quimioluminescência para detecção de anticorpos anti-HAV. Informações sobre vacinação foram obtidas em base de dados do município. Análises estatísticas incluíram cálculo de proporções, testes qui-quadrado e Teste Exato de Fisher.

**Resultados:**

Das 216 amostras, 35 (16,2%; IC95% 11,9; 21,7) foram reagentes para anti-HAV IgG, indicando imunidade prévia; nenhuma amostra apresentou IgM reagente. A prevalência de IgG entre não vacinados foi de 4,6% e de 11,6% entre os vacinados. Dos 25 casos reagentes previamente vacinados, todos estavam na faixa etária de 10-14 anos. Dos dez reagentes por exposição natural, cinco estavam na faixa de 10-14 anos e cinco na faixa de 15-19 anos. Não houve diferença significativa na soroprevalência de IgG por sexo (p-valor 0,685) ou por escola (p-valor 0,078).

**Conclusão:**

A soroprevalência de anti-HAV IgG em adolescentes de Curitiba foi baixa, com predominância de imunidade vacinal em relação à adquirida por infecção natural, e ausência de casos de infecção aguda.

## Resumen

**Objetivo:**

Estimar la seroprevalencia de anticuerpos contra el Virus de la Hepatitis A (anti-VHA) -Inmunoglobulina G (IgG) e Inmunoglobulina M (IgM)- en adolescentes estudiantes de Curitiba, Paraná (Brasil), en el contexto de un brote e identificar la proporción de infecciones naturales e inmunidad a la vacuna.

**Métodos:**

Estudio transversal basado en una encuesta serológica realizada entre el 3 y el 5 de julio de 2024 en cinco escuelas del estado. La muestra se estratificó de manera aleatoria proporcional al número total de estudiantes por escuela. Se incluyeron a adolescentes de entre 10 y 19 años de edad, con consentimiento individual y de los tutores legales. La recolección se realizó en las escuelas, y las muestras se analizaron por quimioluminiscencia para la detección de anticuerpos anti-VHA. La información sobre la vacunación se obtuvo de la base de datos del municipio. Los análisis estadísticos incluyeron el cálculo de proporciones, pruebas de chi-cuadrado y la prueba exacta de Fisher.

**Resultados:**

De las 216 muestras, 35 (16,2%; IC 95% 11,9; 21,7) fueron reactivas para anti-VHA IgG, lo que indica inmunidad previa; ninguna muestra obtuvo IgM reactivo. La prevalencia de IgG entre los no vacunados fue del 4,6%; y del 11,6% entre los vacunados. De los 25 casos de reactivos vacunados previamente, todos estaban en el grupo de edad de entre 10-14 años. De los diez reactivos por exposición natural, cinco estaban en el rango de edad de entre 10-14 años; y cinco, en el rango de edad de entre 15-19 años. No se encontraron diferencias significativas en la seroprevalencia de IgG por sexo (p-valor 0,685) o por escuela (p-valor 0,078).

**Conclusión:**

La seroprevalencia de anti-VHA IgG en adolescentes de Curitiba fue baja, con predominio de inmunidad vacunal con relación a la adquirida por infección natural, y ausencia de casos de infección aguda.

**Aspectos éticos**

**Esta pesquisa respeitou os princípios éticos, obtendo os seguintes dados de aprovação:**

Comitê de ética em pesquisa: Comissão Nacional de Ética em Pesquisa

Número do parecer: 7.545.013

Data de aprovação: 13/6/2025

Certificado de apresentação de apreciação ética: 87956525.0.0000.0008

Registro do consentimento livre e esclarecido: Não se aplica.

## Introdução

A hepatite A é uma doença infecciosa do fígado causada pelo Vírus da Hepatite A (HAV), com transmissão primária pela via fecal-oral, seja por contato direto ou pelo consumo de água e alimentos contaminados (1). O HAV é notavelmente resistente no ambiente, podendo permanecer infeccioso por horas em mãos, dias em alimentos e meses em superfícies e fezes (2). Embora autolimitada e geralmente benigna em crianças, a infecção pode apresentar maior gravidade em adolescentes e adultos, com risco aumentado de desfechos graves como hepatite colestática, hepatite fulminante e óbito (3).

Nas últimas décadas, diversos países, incluindo o Brasil, têm testemunhado uma transição epidemiológica da hepatite A (4,5,6,7). A melhoria nas condições de saneamento e a vacinação precoce tem reduzido a exposição viral em idades mais jovens, levando a um aumento da suscetibilidade em adolescentes e jovens adultos. Nesse cenário, a vacinação para crianças e jovens adultos é apontada como uma ferramenta fundamental de saúde pública para a prevenção de surtos (8).

No Brasil, entre 2007 e 2016, houve um declínio na incidência de hepatite A em todas as regiões, particularmente no grupo com menos de 20 anos (9). Contudo, essa tendência foi revertida após 2016, com um aumento na incidência observado especificamente em homens de 20 a 39 anos nas regiões Sul e Sudeste do país (9). A comparação entre os anos de 2014 e 2024 demonstra uma redução de 99,9% na taxa de incidência de hepatite A, tanto em crianças menores de 5 anos quanto no grupo de 5 a 9 anos (10). As regiões Nordeste (29,2%) e Norte (24,5%) historicamente concentram mais da metade de todos os casos confirmados de hepatite A no Brasil, no período de 2000 a 2024. Contudo, a tendência da incidência tem mostrado alterações em outras regiões, como a elevação registrada na região Sudeste em 2017 e 2018 e de 2022 a 2024. Esse cenário é reforçado pela alta incidência observada em algumas capitais do Sul e Sudeste em 2024, com destaque para Curitiba e Porto Alegre (10).

Em 2023, o município de Curitiba, Paraná, vivenciou um surto de hepatite A com casos concentrados entre jovens adultos e, posteriormente, acometendo adolescentes escolares (11). Diante do surgimento de casos em escolares e da possibilidade de circulação silenciosa do vírus nesse grupo etário, tornou-se necessário compreender a magnitude da exposição prévia ao HAV por meio de um inquérito sorológico. 

Hipotetizou-se que, com a disponibilização da vacina na rede pública desde 2014 para crianças com idade entre os 12 meses e 5 anos incompletos (4 anos, 11 meses e 29 dias), a prevalência de anticorpos contra o Vírus da Hepatite A (Imunoglobulina G - IgG) poderia ser maior entre os adolescentes mais jovens (10-14 anos) que podem ter sido contemplados na estratégia de vacinação na rede pública em comparação com os mais velhos (15-19 anos). Assume-se que ambos os grupos estão inseridos em um contexto sanitário com bom saneamento básico, o que reduziria a possibilidade de exposição natural. 

Este estudo teve como objetivo estimar a soroprevalência de anticorpos contra o Vírus da Hepatite A (anti-HAV), Imunoglobulina G (IgG) e Imunoglobulina M (IgM), em adolescentes escolares de Curitiba, Paraná, em 2024, no contexto de um surto, e identificar a proporção de infecções naturais e de imunidade vacinal.

## Métodos

### Delineamento

Estudo transversal com base em um inquérito sorológico realizado entre os dias 3 e 5 de julho de 2024, em cinco escolas estaduais do município de Curitiba, Paraná, aqui denominadas Escola A, Escola B, Escola C, Escola D e Escola E. 

### Participantes

A população de estudo foi composta por adolescentes regularmente matriculados nas instituições selecionadas. A abordagem dos alunos e responsáveis legais foi intermediada pelas diretorias das escolas, em alinhamento com o Núcleo Regional de Educação de Curitiba. A coleta de sangue foi realizada por equipe da Secretaria Municipal da Saúde, nas próprias escolas, mediante consentimento informado. 

### Critérios de elegibilidade

Foram incluídos estudantes com consentimento individual e dos responsáveis. Foram excluídos aqueles cuja amostra laboratorial foi insuficiente ou inapropriada para teste (volume insuficiente, hemólise ou rompimento da cadeia de preservação).

### Vieses

Consideraram-se como potenciais fontes de viés os de seleção, informação e não resposta. O viés de seleção pode ter ocorrido em função do tamanho amostral final inferior ao estimado e de recusas na participação, minimizado pela seleção aleatória estratificada proporcional por escola e ampliação do número de convidados. O viés de informação pode decorrer de registros incompletos de vacinação ou falhas na classificação laboratorial, reduzido pelo uso de bases oficiais de imunização e análises sorológicas por método padronizado em laboratório de referência com controle de qualidade. 

### Tamanho do estudo

Foram selecionadas cinco escolas por conveniência: quatro delas por terem registrado casos confirmados hepatite A entre 1º de novembro de 2023 e 29 de maio de 2024, e uma (Escola E), sem casos confirmados, foi incluída para comparação. A Escola E situa-se em distrito sanitário da região sul de Curitiba caracterizado por indicadores socioambientais menos favoráveis, nomeadamente água e saneamento (12).

**Figura 1:**
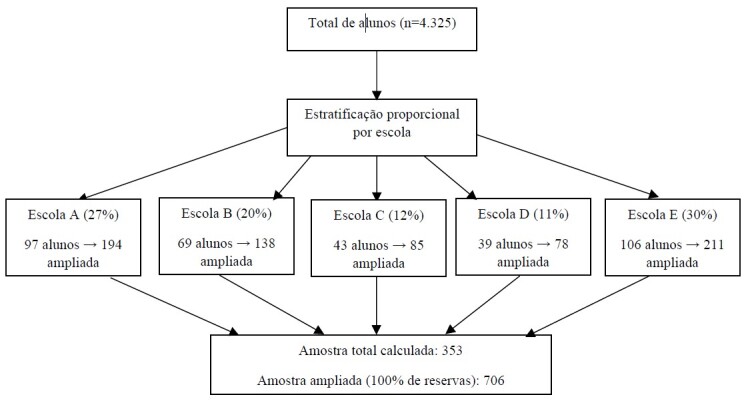
Distribuição da amostra calculada por escola segundo amostragem aleatória estratificada proporcional ao total de alunos. Curitiba, Paraná, 2024

Foi realizada uma amostragem aleatória estratificada proporcional ao total de alunos por escola, com base em uma população de 4.325 estudantes. Considerando uma frequência esperada de 50% e erro de 5%, a amostra estimada foi de 353 alunos. Para compensar possíveis perdas, foram convidados a participar o dobro de alunos (n=706), que foram selecionados aleatoriamente, respeitando a proporção por escola. A Figura 1 detalha o processo da referida amostragem.

### Variáveis

As variáveis analisadas incluíram sexo, idade e escola, bem como o resultado sorológico (reagente ou não reagente) e a situação vacinal (vacinado ou não vacinado). Todas as informações dos participantes foram registradas em fichas pelo mesmo profissional responsável pela coleta de sangue. Para análise de diferença de resultados entre faixas etárias, os participantes foram divididos em dois grupos: 10-14 anos e 15-19 anos. 

### Fontes de dados

Utilizou-se a base de alunos matriculados nas escolas incluídas para as variáveis sexo, idade, escola e a base de dados de imunização de Curitiba, Paraná, para status vacinal para hepatite A. Os referidos dados de imunização são preenchidos localmente no sistema e-Saúde e carregados no Sistema de Informações do Programa Nacional de Imunizações. Para os resultados sorológicos, as amostras de soro foram analisadas por quimioluminescência para detecção de anticorpos contra o Vírus da Hepatite A (Imunoglobulina G e Imunoglobulina M) utilizando kits da Abbott. Os resultados foram comunicados individualmente aos participantes via aplicativo Saúde Já Curitiba. 

### Análises estatísticas

Frequências descritivas foram realizadas para caracterizar a amostra. Foram calculadas proporções e prevalências com respectivos intervalos de confiança (IC) pelo score de Wilson. O intervalo de confiança foi estimado apenas para a prevalência geral, devido à ausência de informação completa sobre o número total de não vacinados por escola. Para comparações entre grupos, utilizaram-se os testes qui-quadrado e Exato de Fisher, conforme aplicável, adotando-se nível de significância de 5% (p-valor<0,05). As análises foram conduzidas no software R versão 4.4.0, Epi Info7.2.6.0 e Microsoft Excel 2016. 

## Resultados

Foram testadas 216 amostras de alunos das escolas selecionadas, apesar do tamanho amostral calculado ter sido 353. A idade dos participantes variou de 10 a 19 anos, com média de 13,8 anos (desvio padrão=2,3). A Tabela 1 apresenta as características demográficas dos adolescentes testados.

**Tabela 1:** Características demográficas de adolescentes testados para anticorpos contra o Vírus da Hepatite A - Imunoglobulina G. Curitiba, Paraná, 2024 (n=216)

Característica	n (%)
Faixa Etária (anos)	
10-12	42 (19,4)
13-15	84 (38,9)
16-18	78 (36,1)
19	12 (5,6)
**Sexo**	
Feminino	127 (58,8)
Masculino	89 (41,2)
**Escola**	
Escola A	52 (24,1)
Escola B	35 (16,2)
Escola C	35 (16,2)
Escola D	30 (13,9)
Escola E	64 (29,6)

Das amostras testadas, 35 (16,2%; IC95% 11,9; 21,7) foram reagentes para anticorpos contra o Vírus da Hepatite A - Imunoglobulina G (anti-HAV IgG). Nenhuma amostra apresentou resultado reagente para a Imunoglobulina M (Tabela 2). 

A prevalência geral de anti-HAV IgG entre os sexos não apresentou diferença estatisticamente significativa (p-valor 0,685). Após excluir as amostras de indivíduos previamente vacinados contra a hepatite A, a prevalência de anti-HAV IgG entre os não vacinados foi de 4,6% e de 11,6% entre os vacinados (Tabela 2). 

**Tabela 2:** Resultado do teste para anticorpos contra o Vírus da Hepatite A - Imunoglobulina G por sexo e escola. Curitiba, Paraná, 2024 (n=216)

Escola	Amostra calculada	Testes realizados	IgG^a^ reagente		Prevalência geral (IC95%^b^)	Casos IgG reagentes em não vacinados	Prevalência em não vacinados (%)
			Masc.	Fem.			
Escola A	97	52	6	8	26,9 (16,77; 40,25)	3	5,7
Escola B	69	35	3	3	17,1 (8,10; 32,68)	4	11,4
Escola C	43	35	3	2	14,3 (6,26; 29,38)	0	0,0
Escola D	39	30	1	0	3,3 (0,59; 16,67)	1	3,3
Escola E	106	64	3	6	14,1 (7,58; 24,62)	2	3,1
Total	353	216	16	19	16,2 (11,89; 21,70)	10	4,6

a IgG: Imunoglobulina G; ^b^IC95%: intervalo de confiança de 95%.

**Figura 2:**
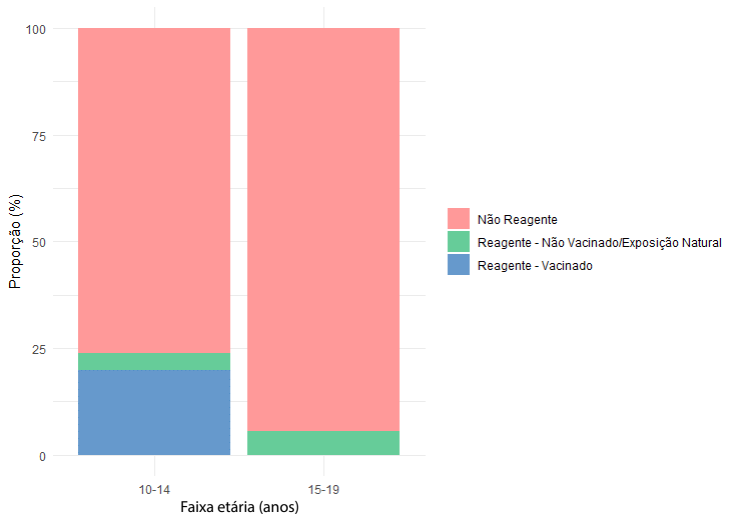
Distribuição por faixa etária do status sorológico e vacinal para o Vírus da Hepatite A. Curitiba, Paraná, 2024 (n=216)

Para análise de diferença entre faixas etárias, a amostra foi dividida em dois grupos: de 10-14 anos e de 15-19 anos. A Figura 2 ilustra a distribuição do status sorológico e vacinal por faixa etária. Observou-se que os anticorpos anti-HAV IgG reagentes foram mais frequentes entre adolescentes vacinados da faixa etária de 10-14 anos, diferença que foi estatisticamente significativa (p-valor<0,001). Entre os não vacinados, a distribuição dos reagentes foi semelhante entre as duas faixas etárias (p-valor 0,744), não indicando diferença entre grupos. 

Não houve diferença na prevalência de IgG reagente para o Vírus da Hepatite A entre as escolas participantes (p-valor 0,078). Quando comparada isoladamente a escola localizada em distrito sanitário mais vulnerável, sem casos confirmados de hepatite A (Escola E, 9/55 reagentes), com o conjunto das demais escolas que registraram casos (26/126 reagentes), também não se observou diferença estatisticamente significativa (p-valor 0,724).

## Discussão

Este estudo evidenciou baixa soroprevalência de anticorpos contra o Vírus da Hepatite A entre adolescentes escolares de Curitiba, sem evidência de infecção aguda no momento do inquérito, mesmo em contexto de surto recente no município. Os achados indicam circulação viral limitada nessa população e sugerem que a imunidade observada decorre predominantemente da vacinação, especialmente entre adolescentes mais jovens. 

Algumas limitações devem ser consideradas na interpretação dos resultados. A seleção das escolas por conveniência e a taxa de resposta inferior à estimada podem ter introduzido viés de seleção, restringindo a generalização dos achados para todos os adolescentes do município. Além disso, possíveis falhas nos registros de vacinação podem ter resultado em erro de classificação do status vacinal. A ausência de variáveis socioeconômicas também limita a avaliação de determinantes contextuais associados à exposição ao vírus e à adesão à vacinação.

A soroprevalência observada foi inferior àquela previamente descrita em estudos conduzidos em Curitiba, Paraná em diferentes períodos, os quais reportaram prevalências que variaram entre 34,0% (2021) e 39,0% (2011) (13,14). Essa redução é compatível com o cenário de transição epidemiológica da hepatite A, caracterizado pela diminuição da exposição natural ao vírus em contextos de melhoria das condições de saneamento e ampliação da vacinação infantil. No Brasil, a introdução da vacina contra hepatite A no Programa Nacional de Imunizações (PNI) para crianças de 12 meses a 5 anos incompletos, a partir de 2014, tem sido associada à queda sustentada da circulação viral e ao deslocamento da suscetibilidade para faixas etárias mais velhas (5,15,16).

A ausência de diferenças estatisticamente significativas na soroprevalência entre as escolas participantes, inclusive naquela situada em distrito sanitário com indicadores socioambientais menos favoráveis, aponta para uma distribuição relativamente homogênea da imunidade nesse conjunto de adolescentes. Esse achado sugere que, no contexto avaliado, a proteção conferida pela vacinação pode ter atenuado desigualdades territoriais previamente associadas à transmissão da hepatite A, padrão já descrito em crianças de grupos socioeconômicos desfavorecidos (17). 

A maior prevalência de anticorpos entre adolescentes vacinados de 10 a 14 anos é consistente com a faixa etária contemplada pela introdução da vacina contra hepatite A no PNI, podendo ser efeito de coorte vacinal. Em contraste, os adolescentes de 15 a 19 anos, que não integraram a coorte inicialmente elegível para vacinação sistemática, apresentaram menor prevalência de anticorpos. Evidências consistentes com esse padrão têm sido documentadas no Brasil após a introdução da vacina contra hepatite A, com expressiva redução da incidência da doença em crianças, especialmente nas faixas etárias diretamente contempladas pela imunização, e diminuição sustentada da circulação viral no país, mesmo em um contexto de cobertura vacinal heterogênea entre os estados (18,19).

Em síntese, a soroprevalência de anticorpos contra o Vírus da Hepatite A entre adolescentes escolares de Curitiba foi baixa, sem evidência de infecção aguda no período avaliado. A maior imunidade observada entre adolescentes mais jovens vacinados sugere efeito da vacinação infantil na redução da suscetibilidade populacional. Esses achados destacam a importância da vigilância sorológica contínua para monitorar lacunas de imunidade e subsidiar estratégias de prevenção em contextos de mudança do perfil epidemiológico da hepatite A.

## Data Availability

O banco de dados anonimizado e utilizado nesta pesquisa está disponível publicamente no repositório SciELO Data, vinculado à revista Epidemiologia e Serviços de Saúde (RESS), e pode ser acessado por meio do link: https://doi.org/10.48331/SCIELODATA.DM2G9A. O conjunto de dados deve ser referenciado conforme a citação correspondente no repositório.
