# Supplementary material for: Soroprevalência de hepatite A em adolescentes escolares: estudo transversal em Curitiba, Paraná, 2024
Source: Epidemiol Serv Saude. 2026 Feb 9;35:e20250870. [Article in Portuguese] doi: 10.1590/S2237-96222026v35e20250870.pt (PMC12888087; doi:10.1590/S2237-96222026v35e20250870.pt)
Supplement: Translation

# Translation: Seroprevalence of hepatitis A in adolescent students: A cross-sectional study in Curitiba, Paraná, 2024

**DOI:** 10.1590/S2237-96222026v35e20250870.en

**Authors:** Janilza Silveira Silva, Renata Barbosa Vilaça Marques de Carvalho, Tatiane Bartneck Telles, Alcides Souto de Oliveira, Leia Regina Silva, Liza Regina Bueno Rosso, Diego Spinoza dos Santos, Clea Elisa Ribeiro, Monique Boese, Katiuska Ferraz Jansen Negrello, Cláudia Weingaertner Palm, Dirlene Pacheco Venâncio, Aroldo José Borges Carneiro, Silvio Luis Rodrigues Almeida

**Affiliations:** 1 Ministério da Saúde, Programa de Treinamento em Epidemiologia Aplicada aos Serviços do SUS, Brasília, DF, Brazil; 2 Prefeitura de Curitiba, Secretaria Municipal da Saúde, Paraná, PR, Brazil; 3 Centro de Epidemiologia da Secretaria Municipal da Saúde de Curitiba, Paraná, PR, Brazil; 4 Centro de Informações Estratégicas em Vigilância em Saúde de Curitiba, Paraná, PR, Brazil

**Keywords:** Hepatitis, Virus Hepatitis A, Adolescent, Disease Outbreak, Seroepidemiological Studies.

## Abstract

**Objective:**

Estimate the seroprevalence of antibodies against Hepatitis A Virus (anti-HAV)-Immunoglobulin G (IgG) and Immunoglobulin M (IgM)-among adolescent students in Curitiba, Paraná, in the context of an outbreak, and identify the proportion of natural infections and vaccine immunity.

**Methods:**

A cross-sectional study based on a serological survey was conducted between July 3 and 5, 2024 in five state schools. Stratified random sampling was proportional to the total number of students per school. Adolescents aged 10-19 years were included with individual and legal guardian consent. Collection was conducted in the schools and the samples were analyzed by chemiluminescence for anti-HAV antibodies detection. Information on vaccination was obtained from the municipality’s database. Statistical analyses included calculation of proportions, chi-square tests, and Fisher’s exact test.

**Results:**

Of the 216 samples, 35 (16.2%; 95%CI 11.9; 21.7) were reactive for anti-HAV IgG, indicating previous immunity; none of the samples showed reactive IgM. IgG prevalence among the unvaccinated was 4.6% and 11.6% among the vaccinated. Of the 25 previously vaccinated reactive cases, all were in the 10-14 years age group. Of the ten reactive by natural exposure, five were in the 10-14 years age group and five in the 15-19 years age group. IgG seroprevalence showed no significant difference by gender (p-value 0.685) or school (p-value 0.078).

**Conclusion:**

Seroprevalence of anti-HAV IgG among adolescents in Curitiba was low, predominating vaccine immunity over that acquired by natural exposure, and absence of acute infection cases.

**Ethical aspects**

**This research respected the ethical principles, having obtained the following approval data:**

Research Ethics Committee: National Committee for Research Ethics

Opinion number: 7.545.013

Approval date: 13/6/2025

Certificate of Submission to Ethical Appraisal: 87956525.0.0000.0008

Informed consent: Not applicable.

## Introduction

Hepatitis A is an infectious liver disease caused by the Hepatitis A Virus (HAV), primarily transmitted via the fecal-oral route either through direct contact or by consuming contaminated water and food (1). HAV is remarkably resistant in the environment and can remain infectious for hours on hands, days on food, and months on surfaces and feces (2). Self-limiting and usually benign in children, the infection may be more severe in adolescents and adults, with an increased risk of serious outcomes such as cholestatic hepatitis, fulminant hepatitis, and death (3).

In recent decades, several countries, including Brazil, have witnessed an epidemiological transition from hepatitis A (4,5,6,7). Improved sanitation conditions and early vaccination have reduced viral exposure at younger ages, leading to increased susceptibility among adolescents and young adults. In this scenario, vaccination for children and young adults is highlighted as a fundamental public health tool for preventing outbreaks (8).

From 2007 to 2016, Brazil recorded a decline in hepatitis A incidence in all country regions, particularly among those under 20 years old (9). But this trend reversed after 2016 due to increased incidence observed specifically among men aged 20-39 years in Southern and Southeastern Brazil (9). Comparison between 2014 and 2024 data shows a 99.9% reduction in the hepatitis A incidence rate, both in children under 5 and in the 5-9 years age group (10). States from the Northeastern (29.2%) and Northern (24.5%) regions historically concentrate over half of all confirmed hepatitis A cases in Brazil recorded from 2000 to 2024. However, the incidence trend has shown changes in other regions, with increasing cases recorded in the Southeast between 2017 and 2018 and from 2022 to 2024. Such scenario gets reinforced by the high incidence observed in some South and Southeast state capitals in 2024, especially Curitiba and Porto Alegre (10).

In 2023, the city of Curitiba, Paraná State, experienced an hepatitis A outbreak with cases concentrated among young adults and, later, adolescent students (11). Given the emergence of cases in schoolchildren and the possible silent circulation of the virus among this age group, it became necessary to understand the magnitude of previous HAV exposure via a serological survey. 

We posited that given the available vaccine in the public network since 2014 for children aged from 12 months to 5 incomplete years (i.e., 4 years, 11 months and 29 days), the prevalence of antibodies against Hepatitis A virus (Immunoglobulin G - IgG) could be higher among younger adolescents (10-14 years old) who may have been included in the public vaccination strategy compared with their older peers (15-19 years old). We assumed that both groups circulate in healthy contexts with good basic sanitation, which would reduce the possibility of natural exposure. 

Hence, this study estimated the seroprevalence of antibodies against Hepatitis A Virus (anti-HAV)-Immunoglobulin G (IgG) and Immunoglobulin M (IgM)-among adolescent students in Curitiba, Paraná, in 2024 within the context of an outbreak, and identified the proportion of natural infections and vaccine immunity.

## Methods

### Design

A cross-sectional study based on a serological survey was conducted between July 3 and 5, 2024, in five state schools in the city of Curitiba, Paraná, named School A, School B, School C, School D and School E.

### Participants

Study population encompassed adolescents regularly enrolled in the selected institutions. Students and their legal guardians were approached with the mediation of the school boards, in compliance with the Regional Education Center of Curitiba. Blood was collected by a team from the Municipal Health Department in the schools, after informed consent. 

### Eligibility criteria

Students with informed assent and consent by their guardian were included. Those whose laboratory sample was insufficient or inadequate for testing (insufficient volume, hemolysis, or disruption of the preservation chain) were excluded.

### Biases

Potential bias included selection, information and non-response sources. Selection bias may have occurred due to the final sample size, lower than estimated, and refusals to participate which were minimized by the proportional stratified random selection by school and the increase in the number of invited. Information bias may result from incomplete vaccination records or failures in laboratory classification, reduced by the use of official immunization databases and serological analyses by a standardized method in a reference laboratory with quality control. 

### Sample size

Five schools were selected by convenience: four of them for having registered confirmed hepatitis A cases between November 1, 2023 and May 29, 2024, and one (School E) with no confirmed cases was included for comparison. School E is located in a sanitary district of southern Curitiba characterized by less favorable socio-environmental indicators, namely water and sanitation (12).

A stratified random sampling was calculated proportional to the total number of students per school, based on a population of 4,325 students. Considering an expected 50% attendance and a 5% error. the estimated sample was 353 students. To compensate for possible losses, we invited twice as many students to participate (n=706) who were randomly selected, respecting the proportion per school. Figure 1 details the sampling process.

**Figure 1:** Distribution of the calculated sample per school according to stratified random sampling proportional to the total number of students. Curitiba, Paraná, 2024

### Variables

Analyzed variables included gender (male and female), age (age groups) and school as well as serological results (reactive or non-reactive) and vaccination status (vaccinated or unvaccinated). All information was recorded on a form by the same health professional responsible for the blood collection. To analyze the difference in results between age groups, participants were divided into two groups: 10-14 years and 15-19 years. 

### Data sources

The database of students enrolled in the included schools was consulted to collected the variables gender, age, school. The immunization database of Curitiba was consulted to access the vaccination status for hepatitis A. Such immunization data are filled in locally on the e-Health system and uploaded to the National Immunization Program Information System. For serological results, the serum samples underwent chemiluminescence analysis to detect antibodies against Hepatitis A virus (Immunoglobulin G and Immunoglobulin M) using Abbott kits. Results were communicated individually to the participants via the Saúde Já Curitiba app. 

### Statistical analyses

Descriptive frequencies were calculated for sample characterization. Proportions and prevalences with respective confidence intervals (CI) were calculated using the Wilson score. Confidence interval was estimated only for the overall prevalence given the lack of complete information on the total number of unvaccinated students per school. Comparisons between groups used the Chi-squared and Fisher’s exact tests as applicable, adopting a 5% significance level (p-value<0.05). All analyses were performed on R version 4.4.0, Epi Info7.2.6.0 and Microsoft Excel 2016.

## Results

A total of 216 samples from students of the selected schools were tested, although the calculated sample size was 353. Participant age ranged from 10 to 19 years, with a mean age of 13.8 years (SD=2.3). Table 1 summarizes the demographic characteristics of the adolescents tested.

**Table 1:** Demographic characteristics of adolescents tested for antibodies against Hepatitis A Virus - Immunoglobulin G. Curitiba, Paraná, 2024 (n=216)

Characteristic	n (%)
Age group (years)	
10-12	42 (19.4)
13-15	84 (38.9)
16-18	78 (36.1)
19	12 (5.6)
**Gender**	
Female	127 (58.8)
Male	89 (41.2)
**School**	
School A	52 (24.1)
School B	35 (16.2)
School C	35 (16.2)
School D	30 (13.9)
School E	64 (29.6)

Of the tested samples, 35 (16.2%; 95%CI 11.9; 21.7) were reactive for antibodies against the Hepatitis A virus - Immunoglobulin G (anti-HAV IgG). No sample presented a positive result for immunoglobulin M (Table 2). 

Overall prevalence of anti-HAV IgG by gender showed no statistically significant difference (p-value 0.685). After excluding samples from individuals previously vaccinated against hepatitis A, anti-HAV IgG prevalence among the unvaccinated was 4.6% and 11.6% among the vaccinated (Table 2).

**Table 2:** Test result for antibodies against Hepatitis A Virus - Immunoglobulin G by gender and school. Curitiba, Paraná, 2024 (n=216)

School	Calculated sample	Tests performed	IgG^a^ reactive		Overall prevalence (95%CI^b^)	Reactive IgG cases among unvaccinated individuals	Prevalence among unvaccinated individuals (%)
			Male	Female			
School A	97	52	6	8	26.9 (16.77; 40.25)	3	5.7
School B	69	35	3	3	17.1 (8.10; 32.68)	4	11.4
School C	43	35	3	2	14.3 (6.26; 29.38)	0	0.0
School D	39	30	1	0	3.3 (0.59; 16.67)	1	3.3
School E	106	64	3	6	14.1 (7.58; 24.62)	2	3.1
Total	353	216	16	19	16.2 (11.89; 21.70)	10	4.6

a IgG: Immunoglobulin G; ^b^95%CI: 95% confidence interval.

For analysis of inter-group differences regarding age, we divided the sample into two groups: 10-14 years old and 15-19 years old. Figure 2 illustrates the distribution of serological and vaccination status by age group. Reactive anti-HAV IgG antibodies were more frequent among vaccinated adolescents in the 10-14 years age group, a difference that was statistically significant (p-value <0.001). Among unvaccinated individuals, reactive distribution was similar between both age groups (p-value 0.744), indicating no inter-group difference.

**Figure 2:** Distribution per age group of serological and vaccination status for Hepatitis A virus. Curitiba, Paraná, 2024 (n=216)

We observed no difference in the prevalence of IgG reactive for Hepatitis A Virus among participating schools (p-value 0.078). When comparing the school located in the most vulnerable health district, with no confirmed hepatitis A cases (School E, 9/55 reactive samples), against the other schools with recorded cases (26/126 reactive samples), we observed no statistically significant difference (p-value 0.724).

## Discussion

Our study observed low seroprevalence of antibodies against the Hepatitis A virus among adolescent students in Curitiba, with no evidence of acute infection at the time of survey, even in the context of a recent outbreak in the city. These findings indicate limited viral circulation among this population and suggest that the immunity observed is predominantly due to vaccination, especially among younger adolescents. 

Certain limitations should be considered in interpreting the results. School selection by convenience and a response rate lower than estimated may have introduced selection bias, restricting the generalization of our findings to all adolescents in the municipality. Moreover, possible errors in vaccination records may have resulted in an misclassification of vaccination status. Lack of socioeconomic variables also limits our assessment of contextual determinants associated with exposure to the virus and adherence to vaccination.

The seroprevalence observed was lower than that previously described in previous studies, which reported prevalences ranging from 34.0% (2021) to 39.0% (2011) (13,14). This reduction is consistent with the epidemiological transition scenario for hepatitis A, characterized by decreased natural exposure to the virus in contexts of improved sanitation conditions and expanded childhood vaccination. In Brazil, the inclusion of the hepatitis A vaccine in the National Immunization Program (PNI) for children aged 12 months to 5 years old, starting in 2014, has been associated with a sustained drop in viral circulation and a shift in susceptibility to older age groups (5,15,16).

The absence of statistically significant differences in seroprevalence among participating schools, including those located in health districts with less favorable socioeconomic indicators, points to a relatively homogeneous distribution of immunity among this group of adolescents. This finding suggests that, in the context evaluated, the protection conferred by vaccination may have mitigated territorial inequalities previously associated with hepatitis A transmission, a pattern already described among children from disadvantaged socioeconomic groups (17). 

Higher prevalence of antibodies among vaccinated adolescents aged 10 to 14 years is consistent with the age group covered by the hepatitis A vaccine included in the PNI and may be a vaccine cohort effect. Conversely, adolescents aged 15 to 19 years, who were not included in the cohort initially eligible for routine vaccination, exhibited a lower prevalence of antibodies. Evidence consistent with this pattern has been documented in Brazil following inclusion of the hepatitis A vaccine, with a significant reduction in its incidence among children, especially in the age groups directly covered by immunization, and a sustained decrease in viral circulation in the country despite the heterogeneous vaccination coverage between states (18,19).

In summary, the seroprevalence of antibodies against the Hepatitis A virus among school adolescents in Curitiba was low, with no evidence of acute infection during the period evaluated. The greater immunity observed among vaccinated younger adolescents suggests the effect of childhood vaccination in reducing population susceptibility. These findings highlight the importance of continuous serological surveillance to monitor immunity gaps and support prevention strategies following changes in the epidemiological profile of hepatitis A.

## Data Availability

The anonymized database used in this research is publicly available at the SciELO Data repository, linked to the journal Epidemiologia e Serviços de Saúde (RESS), and can be accessed on: https://doi.org/10.48331/SCIELODATA.DM2G9A. The dataset should be referenced according to the corresponding citation in the repository.
